# Frequency of bone graft in implant surgery

**DOI:** 10.1186/s40902-016-0064-2

**Published:** 2016-03-31

**Authors:** Hyun-Suk Cha, Ji-Wan Kim, Jong-Hyun Hwang, Kang-Min Ahn

**Affiliations:** 1 Department of Prosthodontics, College of Medicine, University of Ulsan, Asan Medical Center, Seoul, Korea; 2 Department of Oral and Maxillofacial Surgery, College of Medicine, University of Ulsan, Asan Medical Center, 88, Olympic-ro, 43-gil, Songpa-gu, Seoul, 138-736 Korea

**Keywords:** Bone graft, Dental implant, Guided bone regeneration, Sinus lifting, Block bone graft

## Abstract

**Background:**

Implant surgery has become popular with the advance of surgical techniques such as sinus lifting, guided bone regeneration, and block bone graft. However, there were no data about the frequency of bone graft during implant surgery. The purpose of this study was to report the frequency and types of bone graft depending on dental implant patients’ profile to complement the database regarding implant surgery.

**Methods:**

The implant operations had been performed from January 2006 to October 2014. The upper and lower jaws were divided into six sextants. A total of 792 sextants were included in this study. Patient information including sex, age, sites, bone graft, and types of bone were investigated.

**Results:**

A total of 1512 implants had been placed. Male and female sextants were 421 and 371, respectively (M:F = 1:0.88). Average age was 54.3 (ranging from 20 to 88 years old). Implants were placed in the posterior maxilla (322 sextants, 40.7 %), posterior mandible (286 sextants, 36.1 %), anterior maxilla (127 sextants, 16.1 %), and anterior mandible (57 sextants, 7.2 %). Bone graft was performed in 50.3 % of the sextants. Among the bone grafted sites, sinus lifting with lateral approach (22.1 %) and guided bone regeneration (22.7 %) were performed most frequently.

**Conclusions:**

Bone graft in implant surgery was necessary to augment defects. More than half of the sextants needed bone graft for implant installation.

## Background

Dental implant restoration has been considered to be one of the most reliable methods for treating partial or full edentulism [1–3]. Implant surgeries were performed in specialized clinics in the past. Today, however, it is quite popular in general dentistry. Health insurance coverage for dental implant treatment varies from country to country [4–7]. Since 2014, the Korean National Health Insurance has offered partial insurance coverage benefits in elderly patients over 70 for up to two implants and the age limit will be lowered to 65 years old starting from July 2016. Patients need to pay only half of the total fee for two implants. As a result, the demand for dental implant surgery is expected to grow rapidly. However, it is hard to estimate the number of annually placed implants because dental implant surgery was not registered in the national health care system. The frequency of dental implant surgery and the number of fixtures annually placed in Korea can be estimated based on the data from implant companies, which is around 500,000~800,000. However, the data does not show the exact number of dental implants per different sites, i.e., the upper and lower jaws and the frequency of bone graft.

Bone graft is frequently accompanied with dental implant surgery [1, 8]. Various types of bone graft materials are used such as the autogenous bone, allogenic bone, xenogenic bone, and synthetic materials. The most frequently used surgical methods for bone grafts are guided bone regeneration (GBR), block bone graft (BBG), sinus lifting via lateral window, and bone-added osteotome technique. GBR procedure needs bone graft materials and a membrane for selective occlusiveness. Bone graft materials can be used solely or mixed together in different proportions [9]. Similar cases of bone defects can be treated differently according to the surgeon’s preference. Compared with dental implant surgery, bone graft procedures are not covered by the national insurance system. To be registered in the insurance system, basic data on the frequency of bone graft during implant surgery is required. However, there is no data regarding how frequently bone grafts are needed during implant surgery and what kinds of graft materials are used. The purpose of this study is to review patient information on bone graft in implant surgery, so that it can be utilized as basic data for standardization of dental implant procedures.

## Methods

This study included patients who visited the Oral and Maxillofacial Department for dental implant from January 2006 to October 2014. The Institutional Review Board (IRB) of our institution issued an exemption and approved the study to use patient information since the subjects are not identifiable, directly or through the data listed in the study. The study was conducted in accordance with the ethical principles provided by the Declaration of Helsinki and the principles of good clinical practice. Patient’s consent forms were not obtained because the study involved retrospective reviews.

Implant operations were performed by one experienced oral and maxillofacial surgeon. Patients were referred from the Department of Prosthodontics. The sites for bone graft were divided into six parts according to the anterior and posterior teeth. Thus, full edentulous maxilla restored with full implant-fixed prosthodontics was considered to have three sextants.

Bone grafts were classified according to the types of procedures (GBR, sinus lifting, and autogenous BBG) and types of bone used (autogenous bone, allogenic bone, xenogenic bone, and synthetic materials). BBG was subdivided according to the source of the bone such as the ramus, chin, anterior nasal spine (ANS), maxillary tuberosity (MT), canine space (CS), and iliac bone.

## Results

A total of 792 sextants were included in this study. The numbers of male and female patients were 421 and 371, respectively. Average age was 54.3 ± 13.3 (from 20 to 88 years old). A total of 1512 implants were installed. Implants were placed in the posterior maxilla (322 sextants, 40.7 %), posterior mandible (286 sextants, 36.1 %), anterior maxilla (127 sextants, 16.1 %), and anterior mandible (57 sextants, 7.2 %).

Bone graft was performed in 50.3 % of all sextants (Table 1). The anterior maxillary area required bone graft most frequently (77.2 %). Bone graft in the posterior maxilla was performed using three different techniques such as sinus lifting via lateral approach or osteotome (175 sextants, 54.3 %), GBR (22 sextants, 6.8 %), and BBG (5 sextants, 1.6 %).

**Table 1:** Type of bone graft procedures and frequencies in each sextant

	Ant Mx	Post Mx	Ant Mn	Post Mn	Total	Percent
No BG	29	120	50	195	394	49.7
GBR	72	22	3	83	180	22.7
SL + BAO	0	175	0	0	175	22.1
BBG	26	5	4	8	43	5.4
Total	127	322	57	286	792	100.0
% of BG	77.2	62.7	12.3	31.8	50.3	100.0

*Mx* maxilla, *Mn* mandible, *Post* posterior, *BG* bone graft, *GBR* guided bone regeneration, *SL* sinus lifting, *BAO* bone-added osteotome, *BBG* block bone graft

Sinus lifting (22.1 %) and GBR (22.7 %) procedures were the two most common bone graft methods in the overall sextants. During the GBR procedure, xenogenic bone was used the most frequently with absorbable membrane (Table 2). Bovine bone was the only source for xenogenic bone. Xenogenic bones that were used included Bio-Oss^®^ (Geistlich, Swiss), A-Oss^®^ (Osstem, Korea), B-Oss^®^ (Osstem, Korea), and BBP^®^ (Oscotech, Korea). Membranes for the GBR procedure were all absorbable porcine origin such as Bio-Gide^®^ (Geistlich, Swiss), Collagen^®^ (Genoss, Korea), Oss-guide^®^ (Bioland, Korea), and Rapiderm^®^ (Darim, Korea). During operation, the exposed implant threads were covered with autogenous bone and augmented with xenogenic bone or synthetic materials (Osteon^®^, Genoss, Korea).

**Table 2:** Types of bone used in guided bone regeneration

GBR	Auto only	Auto + xeno	Xeno only	Syn only	Total
No	40	44	79	17	180
%	22.2	24.4	43.9	9.4	100.0

*GBR* guided bone regeneration, *auto* autogenous bone, *xeno* xenogenic bone, *syn* synthetic material, *No* number of sextants

BBG was performed 43 times. Vertical and/or horizontal bone augmentations were the indications for BBG. Donor sites for BBG are listed in Table 3. Ramal bone was the most common donor site for BBG. Only one patient who underwent marginal mandibulectomy for ameloblastoma needed iliac bone graft for implant surgery.

**Table 3:** Donor sites for autogenous block bone graft

	Ramus	Chin	ANS	MT	CS	Iliac	Total
No	19	5	10	6	2	1	43
%	44.2	11.6	23.3	14.0	4.7	2.3	100.0

*ANS* anterior nasal spine, *MT* maxillary tuberosity, *CS* canine space, *No* number of sextants

The frequency of bone graft requirement according to age is listed in Table 4. In their 60s, patients required bone graft most frequently (55.6 %). Due to medical and general health conditions, less frequent bone grafts were performed in patients over 70.

**Table 4:** Age and type of bone graft

Ages	20–29	30–39	40–49	50–59	60–69	70–79	>80	Total
No bone graft	23	34	49	135	88	51	14	394
GBR	9	26	32	53	47	10	3	180
Sinus lifting	1	22	30	59	51	12	0	175
BBG	6	3	7	15	12	0	0	43
Total	39	85	118	262	198	73	17	792
% of BG	41.0	60.0	58.5	48.5	55.6	30.1	17.6	50.3

*Ages* years old, *GBR* guided bone regeneration, *BBG* block bone graft, *BG* bone graft

## Discussion

Dental implant is considered as the most reliable and convenient treatment for partial and full edentulism. Long-term follow-up of the implants showed successful survival rate of over 90 % [10]. Korea is one of the fastest aging countries in the world. The Korean national insurance system covers half of the expenses for up to two implants in patients over 70 since 2014. The age limit will be lowered to 65 in the near future. Therefore, dental implant surgery is expected to become more popular in elderly patients. The statistics about dental implant surgery and bone graft are not reported to the Korean Statistical Information Service (KOSIS). Although estimations can be made based on the data from the implant companies which filed yearly sales report, these statistics do not represent the exact number of implants in the patients’ jaw.

In this study, 50.3 % of the patients required bone graft during implant surgery. The frequency of bone graft is expected to be higher in the Department of Oral and Maxillofacial Surgery than in the local clinics. However, implant surgeons should be prepared for bone graft during implant surgery because more and more complicated patients could visit their clinics. The anterior maxillary area required bone graft more than 77 %. Because of the high esthetic demands in the anterior maxilla, bone augmentation was performed even though there was no bone fenestration or dehiscence [11–13]. Autogenous bone graft in exposed threads of the implant was suggested as a golden standard [14]. After autogenous bone graft, xenogenic bone and absorbable membrane were used for additional augmentation for long-term esthetic results. At least 1.5~2 mm of buccal bone is required for esthetic results in the anterior maxilla [15]. In this study, GBR was performed 72 times in the anterior maxilla. The indications for GBR are dehiscence or fenestration wound or thin labial plate which was expected to resorb during healing. If the width of the residual alveolar bone in the anterior maxilla was less than 3 mm, BBG was performed. ANS bone was commonly grafted to the anterior maxilla in small bone defects. If there is a larger bone defect of more than 1.5 cm, ramal bone was a graft of choice. BBG was performed in the anterior maxilla most frequently than in any other sites.

During GBR procedures, xenogenic bone with/without autogenous bone was the most commonly used. The advantages of the xenogenic bone include slow bone resorption during the healing phase and its wide availability. Although there is no bone dehiscence, xenogenic bone was recommended to graft for the augmentation of the labial bone. In this study, absorbable membrane was used for GBR procedure. There are many kinds of membranes such as collagen, Gore-Tex, titanium, and allogenic dura. The selection of membrane is dependent on the surgeon’s preference. Titanium mesh is well known to preserve bone graft during healing, but it requires open flap to remove mesh and screws. If large sizes of vertical and horizontal defects require augmentation, BBG was chosen instead of GBR procedures.

All block bones were harvested from the intraoral sites except in the case of one patient who needed a large-sized block bone from the iliac to treat mandibular bone defects due to ameloblastoma. BBG is harder to perform compared with the GBR procedure. BBG requires rigid fixation for long-term stability. Screws and miniplates are required for rigid fixation. In our study, ramal bone was harvested most frequently for BBG since it has many advantages. Ramal bone can be harvested in large amounts, and it is easy to harvest. It is an intramembranous bone and it rarely causes complications or changes in facial morphology. The chin bone has been widely used for BBG. However, it has some disadvantages such as numbness in the lower lip, shallowing of the vestibule, morphologic changes, and pulp damage in the anterior mandibular incisors [16]. The chin bone and mandibular body bone were harvested when the bone defects were present in those areas. ANS is a useful site for a small amount of bone graft [17]. When bone defects are less than 1 cm in the anterior maxilla, single-side simple vertical incision is enough to expose ANS. The only caution for ANS is a possible fracture of whole ANS which can cause nasal tip deformity. When bone defects are larger than 1 cm, it is better to harvest bone from the ramus.

Iliac bone graft is usually performed under general anesthesia and thus, this procedure needs hospitalization. It is useful for reconstruction of large-sized bone defects of over 3–4 cm. Resection of benign tumor of the mandible is indication for iliac bone graft. In this study, only one patient underwent iliac bone graft and implant installation. Long-term follow-up showed stable marginal bone preservation with our previous study [8].

MT bone is easy to harvest when extracting the upper third molar. However, the amount of bone is too small and thin to cover large bone defects. Single implant dehiscence or fenestration is the indication for MT bone graft. Canine space is a good source to harvest cortical and marrow bone during implant surgery in the canine and premolar area. Chisel and mallet are enough for harvesting bone from canine space. It can cause postoperative swelling, so pressure dressing is recommended to prevent swelling.

Sinus lifting procedure has been performed for over 40 years with sustained success rates [18]. Autogenous bone graft has been a golden standard for sinus lifting procedure for over two decades. However, there are many studies reporting that xenogenic bone or synthetic materials are enough for sinus bone graft [1, 19, 20]. In this study, sinus lifting had been performed with only xenogenic bone graft. Lateral approach for sinus lifting was performed when the residual alveolar bone is less than 6 mm. When the residual alveolar bone was between 8 and 10 mm, osteotome technique without bone graft was performed.

## Conclusions

In the study, bone graft was necessary to augment the defect area during implant surgery. More than half of the sextants (50.3 %) needed bone graft for implant installation. Anterior maxillary sextant needed bone graft in about 77.2 % cases. GBR was the most commonly performed procedure for bone augmentation.

## References

[1] Cha HS, Kim A, Nowzari H, Chang HS, Ahn KM (2014). Simultaneous sinus lift and implant installation: prospective study of consecutive two hundred seventeen sinus lift and four hundred sixty-two implants. Clin Implant Dent Relat Res.

[2] Kim JH, Kim YK, Bae JH (2013). Retrospective clinical study on sinus bone graft and tapered-body implant placement. J Korean Assoc Oral Maxillofac Surg.

[3] Gonzalez S, Tuan MC, Ahn KM, Nowzari H (2014) Crestal approach for maxillary sinus augmentation in patients with </= 4 mm of residual alveolar bone. Clin Implant Dent Relat Res 16:827-35 10.1111/cid.12067 23557102

[4] Vernazza CR, Rousseau N, Steele JG, Ellis JS, Thomason JM, Eastham J (2015). Introducing high-cost health care to patients: dentists’ accounts of offering dental implant treatment. Community Dent Oral Epidemiol.

[5] Millennium Research G (2002). European markets for dental implants 2001: executive summary. Implant Dent.

[6] Palmqvist S, Soderfeldt B, Arnbjerg D (1991). Subjective need for implant dentistry in a Swedish population aged 45-69 years. Clin Oral Implants Res.

[7] Parker HM, Miller RD (1989). Dental implants and third party carrier coverage. Dent Clin North Am.

[8] Kim A, Kar K, Nowzari H, Cha HS, Ahn KM (2013). Immediate free iliac bone graft after nonsegmental mandibular resection and delayed implant placement: a case series. Implant Dent.

[9] Kim A, Kar K, Nowzari H, Ahn KM, Cha H (2012). Subapical osteotomy to correct dental implant malpositioning and vertical ridge deficiency: a clinical report. J Prosthet Dent.

[10] Ko SH, Chang YY, Um YJ, Jung UW, Kim CS, Cho KS (2009). An 8-year survival rate of immediate implants: retrospective study. J Kor Dent Assoc.

[11] Ribeiro CG, Bittencourt TC, Ferreira CF, Assis NM (2014). An alternative approach for augmenting the anterior maxilla using autogenous free gingival bone graft for implant retained prosthesis. J Oral Implantol.

[12] Chen ST, Buser D (2014). Esthetic outcomes following immediate and early implant placement in the anterior maxilla—a systematic review. Int J Oral Maxillofac Implants.

[13] Wakimoto M, Matsumura T, Ueno T, Mizukawa N, Yanagi Y, Iida S (2012). Bone quality and quantity of the anterior maxillary trabecular bone in dental implant sites. Clin Oral Implants Res.

[14] Buser D, Chappuis V, Bornstein MM, Wittneben JG, Frei M, Belser UC (2013). Long-term stability of contour augmentation with early implant placement following single tooth extraction in the esthetic zone: a prospective, cross-sectional study in 41 patients with a 5- to 9-year follow-up. J Periodontol.

[15] Buser D, Chappuis V, Kuchler U, Bornstein MM, Wittneben JG, Buser R (2013). Long-term stability of early implant placement with contour augmentation. J Dent Res.

[16] Noia CF, Ortega-Lopes R, Ricardo de Albergaria Barbosa J, Barbeiro RH, Mazzonetto R (2012). Evaluation of patients’ perceptions of alterations after chin bone graft harvesting. Implant Dent.

[17] Cho YS, Hwang KG, Park CJ (2013). Postoperative effects of anterior nasal spine bone harvesting on overall nasal shape. Clin Oral Implants Res.

[18] Tatum H (1986). Maxillary and sinus implant reconstructions. Dent Clin N Am.

[19] Markovic A, Misic T, Calvo-Guirado JL, Delgado-Ruiz RA, Janjic B, Abboud M (2015) Two-center prospective, randomized, clinical, and radiographic study comparing osteotome sinus floor elevation with or without bone graft and simultaneous implant placement. Clin Implant Dent Relat Res. doi:10.1111/cid.12373. [Epub ahead of print] 10.1111/cid.12373 26315564

[20] Mertens C, Wiens D, Steveling HG, Sander A, Freier K (2014). Maxillary sinus-floor elevation with nanoporous biphasic bone graft material for early implant placement. Clin Implant Dent Relat Res.

